# Emerging Trends in Arctic Solar Absorption

**DOI:** 10.1029/2021GL095813

**Published:** 2021-12-24

**Authors:** A. Sledd, T. S. L’Ecuyer

**Affiliations:** ^1^ University of Wisconsin‐Madison Madison WI USA; ^2^ Cooperative Institute for Meteorological Satellite Studies Madison WI USA

**Keywords:** Arctic, solar radiation, climate change, clouds, trend detection

## Abstract

Recent satellite observations confirm that the Arctic is absorbing more solar radiation now than at the start of this century in response to declining Arctic sea ice and snow covers. Trends in the solar radiation input to Arctic ocean and land surfaces now each exceed interannual variability at the 95% confidence level, although all‐sky trends have taken 20%–40% longer to emerge compared to clear‐sky conditions. Clouds reduce mean solar absorption and secular trends over both land and ocean, but the effect of clouds on natural variability depends on the underlying surface. While clouds increase the time needed to unambiguously identify trends in nearly all Arctic regions, their masking effects are strongest over oceans. Clouds have extended the time to emergence of already observed clear‐sky trends beyond the existing 21 years Clouds and Earth's Radiant Energy System record in half of eight Arctic seas, supporting the need for continued satellite‐based radiative flux observations over the Arctic.

## Key Points

- Significantly more solar radiation is being absorbed into the Arctic now than at the turn of the century
- Clouds increase the time needed to detect trends in solar absorption
- Clouds exert the largest masking effect over oceans and areas with the greatest sea ice loss

## 1 Introduction

The Arctic is rapidly warming due to increasing greenhouse gases in the atmosphere (Najafi et al., 2015; Overland et al., 2019). Since the beginning of the modern satellite era, the 14 lowest sea ice extent minima have all occurred since 2007 (Scott, 2020), with all seasons experiencing sea ice decline (Stroeve & Notz, 2018). On land, snow cover is also declining, with longer melt seasons (Wang et al., 2018) and a “greening” of the Arctic (Myers‐Smith et al., 2020; Zhu et al., 2016). These changes in surface cover impact surface‐atmosphere heat and moisture exchanges (Serreze & Barry, 2011) creating a fundamentally different environment that has been termed the “new Arctic” (Carmack et al., 2015).

The balance of radiant energy entering and leaving the arctic system is a critical driver of this new Arctic. In equilibrium, incoming solar, or shortwave (SW), radiation at the top of the atmosphere (TOA) is balanced by the sum of solar radiation reflected by the atmosphere and surface and outgoing thermal emission from the Earth. Imbalances owing to changes in absorbed solar radiation can enhance warming locally relative to the global mean. As snow and ice covers recede in response to anthropogenic warming, more solar energy is absorbed at the surface, leading to warmer temperatures that further increase surface melt in processes known as ice and snow‐albedo feedbacks (Curry et al., 1995).

The amount of SW radiation that reaches the surface is, however, strongly modulated by clouds (L’Ecuyer et al., 2019). In the Arctic, clouds' impact on SW is particularly large during summer when the sun shines continuously above the Arctic Circle (Kay et al., 2016; Sedlar et al., 2011). The atmosphere, mainly clouds, accounts for at least two thirds of the planetary albedo (Qu & Hall, 2005). Arctic cloud cover is typically greater than 65% throughout the year (Comiso & Hall, 2014), and this persistent cloud cover reduces interannual variability in the TOA albedo, despite larger year to year variations in sea ice, snow cover, and surface albedo in the new Arctic (Sledd & L’Ecuyer, 2019; Wu et al., 2020). Likewise, the decline in TOA albedo due to ice loss is not as large as it would be if clouds were not present (Pistone et al., 2014; Sledd & L’Ecuyer, 2021).

Changes in SW absorption from the late 1970s to the present have been documented at the surface using various satellite observations and reanalyses (Katlein et al., 2017; Letterly et al., 2018; Perovich et al., 2007; Shi et al., 2010). However, the accuracy of Arctic surface radiative fluxes in reanalyses is often questioned given the challenges of representing Arctic clouds and uncertainties in modeling SW fluxes at the surface (Christensen et al., 2016; Lindsay et al., 2014; Stephens et al., 2012; Tjernström et al., 2008). The launch of the Clouds and Earth's Radiant Energy System (CERES) instruments in 2000 expanded coverage of more direct TOA flux measurements to include polar regions (Loeb et al., 2018). These new observations have been used to quantify trends in SW radiation over the Arctic previously but with limited time periods (Hartmann & Ceppi, 2014; Kato et al., 2006). Further, while some of these studies briefly discuss the influence of cloud cover, none explicitly quantify the influence of clouds on the emergence of SW absorption trends and few consider the role of interannual variability.

Here, we use two decades of CERES observations to document pan‐Arctic solar absorption trends as well as those in individual ocean basins and land regions. The significance of these trends is established via statistical methods that estimate the expected number of years required to observe statistically significant trends relative to interannual (natural) variability. We determine the impact of clouds by comparing solar absorption trends and their time to emergence derived separately from all‐sky and clear‐sky fluxes. The results highlight the rapid changes in energy input to the new Arctic and the role clouds play in shaping regional impacts.

## 2 Materials and Methods

TOA energy balance is defined by the difference between incoming SW (SW *↓*) radiation minus reflected SW (SW *↑*) and emitted longwave radiation. The net SW (SW *↓* ‐ SW *↑*) energy that enters the Arctic system is, therefore, a fundamental driver of the Arctic climate in general and sea ice melt in particular (Choi et al., 2014). This study considers the total SW energy that accumulates in a given year during March through September, which accounts for 95% of incoming solar radiation in the Arctic (Cao et al., 2016). Monthly means of net SW for each grid box *i*, *j* are multiplied by the number of seconds in each month, *t*_*m*_ and summed over March‐September during a single year:

(1) $$SW_{acc_{i,j}}=\sum_{m=3}^{9}{\left(SW^{↓}-SW^{↑}\right)}_{i,j}\times t_{m}.$$

For an individual grid box, ${SW}_{acc_{i,j}}$ has units of Jm^−2^. The total SW_*acc*_ for a region, for example, all land or all ocean, is calculated by multiplying each grid box ${SW}_{acc_{i,j}}$ by its area, *A*_*i*,*j*_, and summing over the region:

(2) $$SW_{acc}=\sum_{i,j}SW_{acc_{i,j}}\times A_{i,j}.$$

This calculation provides the total energy input (unit Joules) for the region.

In addition to being a fundamental driver of Arctic energy balance, SW_*acc*_ is well‐suited to trend and TTE analyses in the Arctic because its time series is stationary once detrended, allowing several statistical methods to be applied. We calculate linear trends using a standard least‐squares linear regression. However, we test the significance of these trends by taking into account the natural variability and autocorrelation present in geophysical variables, a method pioneered by Weatherhead et al. (1998) and Tiao et al. (1990). This method determines if a trend is greater than interannual variations as opposed to simply nonzero as with hypothesis testing. A trend is considered significant at a 95% confidence level if the magnitude is at least twice as large as its standard deviation, $\sigma_{\hat{\omega }}$, estimated by

(3) $$\sigma_{\hat{\omega }}\approx \sigma_{N}{\left[\frac{12dt}{T^{3}}\frac{\left(1+\phi \right)}{\left(1-\phi \right)}\right]}^{1/2},$$

where *σ*_*N*_ is the standard deviation, *T* is the length of the time series, and *dt* is the time interval (*dt* = 1 for annual observations), and *ϕ* is the 1‐lag autocorrelation. The uncertainty is calculated from the detrended anomalies, which are modeled as an autoregressive order one (AR(1)) process. Further details can be found in Weatherhead et al. (1998) and Sledd and L’Ecuyer (2021). This method has been used to study global mean trends in radiation (Phojanamongkolkij et al., 2014) and time to emergence of cloud properties (Chepfer et al., 2018).

This analysis is repeated for individual regions of the Arctic, for which the total SW_*acc*_ energy is calculated in Joules. We create synthetic time series by generating random noise with the same variance and autocorrelation of the detrended time series, and to this noise we add the linear trend determined from the original time series. The variance, autocorrelation, and trends are calculated from 2000–2020, and the synthetic time series are extended to 2300, much longer than is actually required for all trends to emerge. Four hundred time series are generated for a given region to create synthetic ensembles that allow us to predict the mean amount time for trends to emerge (TTE). For trends that are not statistically significant in observations, TTE is meant to roughly guide expectations of how many additional years of observations would be needed to determine the significance of the trend, not to forecast the particular year a trend will emerge.

Accumulated SW is calculated using monthly TOA fluxes from CERES‐EBAF Ed 4.1 over 2000–2020 (Loeb et al., 2018) as presented in the Arctic Observation and Reanalysis Integrate System (ArORIS), a collection of data sets to facilitate studying the Arctic climate (Christensen et al., 2016). Data sets included in ArORIS have been regridded onto a common 2.5*°* x 2.5*°* grid. TOA fluxes in the CERES‐EBAF data set are adjusted using an objective constrainment algorithm so as to be consistent with in situ ocean observations of global heating rates. We calculate SW_*acc*_ using all‐sky fluxes and total‐region clear‐sky fluxes. The latter are calculated in a manner to be consistent with climate models in terms of assumptions of a clear‐sky column as opposed to only being calculated where the footprint is clear of clouds (Loeb et al., 2020). Uncertainty for net SW at the TOA under all‐sky conditions is 3 Wm^−2^ for the Terra‐only period (March 2000–June 2002) and 2.5Wm^−2^ afterward, and under clear‐sky conditions, uncertainty in upwelling SW is 6 Wm^−2^ for the Terra‐only period and 5 Wm^−2^ after (Loeb et al., 2018).

Land and ocean are distinguished using the land fraction included in ArORIS based on the NCEP reanalysis land mask. NCEP land masks from ArORIS are also used to determine the individual land regions. Marginal seas are based on the Multisensor Analyzed Sea Ice Extent regions from the NSIDC interpolated to the ArORIS grid. These regions are mapped in Figure S1 in Supporting Information S1.

## 3 Results

**Figure 1:**
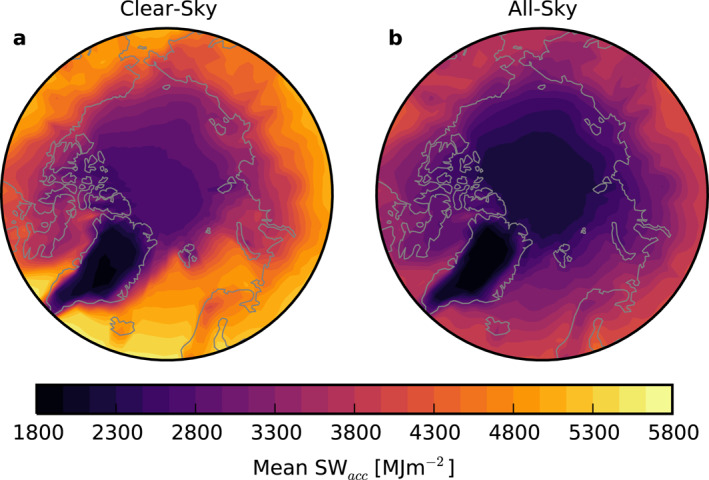
Mean accumulated shortwave (SW_*acc*_) over 2000–2020 from CERES‐EBAF, calculated with top of the atmosphere clear‐sky (a) and all‐sky (b) fluxes.

The 2000–2020 average SW_*acc*_ is shown in Figure 1. Corresponding average sea ice and snow cover for the same period are shown in Figure S2 in Supporting Information S1 using data from Peng et al. (2013) and Hall and Riggs (2015). Without clouds (Figure 1a), regional differences in SW_*acc*_ largely reflect the mean coverage of sea ice and snow (Figures S2a and b in Supporting Information S1). The greatest SW_*acc*_ values, up to 5,600 MJm^−2^, occur over lower latitudes that receive more solar insolation and are consistently free of sea ice. SW_*acc*_ decreases moving poleward in part due to the increasing solar zenith angle, but a sharp transition is visible in the North Atlantic and off the coast of Greenland where sea ice is typically present for at least part of the year. Over the interior Arctic Ocean (>$70^{o}$N), mean SW_*acc*_ ranges from 2000 to 4900 MJm^−2^.

The lowest clear‐sky SW_*acc*_ values are found over the central Greenland ice sheet where the surface is glaciated and relatively bright throughout the year (Stroeve et al., 2013). Regions with high average snow cover on land during the melt season have low SW_*acc*_, including the Canadian Archipelago, western mountains in Norway, and northern coast of Russia. Overall, clear‐sky SW_*acc*_ is similar to SW absorption at the surface (Letterly et al., 2018).

Clouds substantially reduce the magnitude of mean SW_*acc*_ and smooth its spatial heterogeneity (Figure 1b). Mean all‐sky SW_*acc*_ is below 4,600 MJm^−2^ over the entire Arctic, 82% the maximum clear‐sky SW_*acc*_, and the range of mean all‐sky SW_*acc*_ values across the Arctic is about half that of clear‐sky SW_*acc*_. While surfaces with high albedos, for example, perennial sea ice in the central Arctic Ocean and the Greenland ice sheet, exhibit mean SW_*acc*_ lower than the rest of the Arctic, the contrast is substantially reduced relative to clear skies. While the clear‐sky SW_*acc*_ illustrates how the Earth's surface interacts with solar energy in the absence of clouds; the all‐sky SW_*acc*_ actually governs the solar energy input into the Arctic climate system.

**Figure 2:**
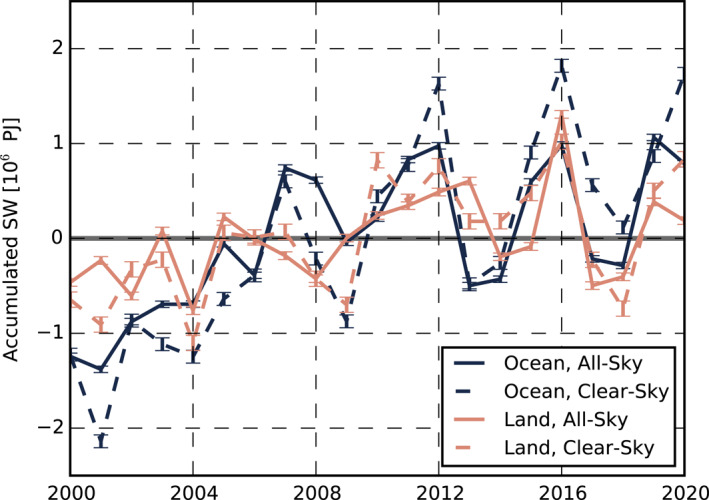
Anomalies of accumulated shortwave over ocean (navy) and land (pink) areas in the Arctic under all and clear‐sky conditions.

The area‐weighted sum of the SW_*acc*_ in Figure 1 gives the total solar energy input into the Arctic system. Given the key role energy imbalances have in driving Arctic climate change, there is considerable interest in whether SW_*acc*_ has systematically changed and where such changes have occurred. Figure 2 shows anomalies of SW_*acc*_ relative to the 2000–2020 mean for both all‐sky (solid lines) and clear‐sky (dashed lines) conditions over land (pink) and ocean (navy). While SW_*acc*_ depends on area, ocean, and land cover nearly equal areas in the Arctic north of 60*°* (Figure S1 in Supporting Information S1). Both clear‐sky and all‐sky SW_*acc*_ trends are greater over ocean than over land (top rows of Table 1), confirming that reductions in sea ice are a stronger driver of surface SW absorption trends than snow cover on land (Letterly et al., 2018). Large positive clear‐sky SW_*acc*_ anomalies occur over the ocean in years with record‐low September sea ice extent, for example, 2007, 2012, 2016, and 2020. All‐sky SW_*acc*_ anomalies are clearly muted in 2012 and 2016 but are comparable to clear‐sky anomalies in 2007 since cloud cover was anomalously low during the 2007 melt season (Kay et al., 2008).

**Table 1:** Characteristics of All‐Sky and Clear‐Sky SW_*acc*_ Radiation Over the Arctic Domains Defined in Figure S1 in Supporting Information S1

	All‐sky					Clear‐sky				
Region	Trend [10^3^ PJ/yr]	Std dev [10^4^ PJ]	SNR [dec^−1^]	Autocor	TTE [yr]	Trend [10^3^ PJ/yr]	Std dev [10^4^ PJ]	SNR [dec^−1^]	Autocor	TTE [yr]
All Arctic^+^	111	84.2	1.3	0.09	16 (4)*	193	108	1.8	0.06	12 (3)*
All Ocean^+^	79.7	56.4	1.4	0.28	16 (4)*	136	65.5	1.9	0.05	11 (3)*
All Land^+^	31.6	42.8	0.73	−0.21	19 (6)*	56.4	49.4	1.1	0.06	17 (5)*
Barents Sea^+^	4.6	8.2	0.58	−0.11	24 (7)	17.0	12.1	1.40	0.22	16 (4)*
Kara Sea^+^	9.96	7.82	1.27	0.22	17 (4)*	21.4	13.6	1.58	0.31	16 (3)*
Laptev Sea^+^	8.32	8.15	1.02	−0.002	17 (5)*	15.3	12.8	1.20	−0.21	14 (4)*
East Siberian Sea^+^	5.82	6.77	0.86	0.014	20 (5)*	10.4	10.5	0.99	−0.13	17 (5)*
Chukchi Sea^+^	6.09	8.18	0.74	0.01	22 (6)*	11.4	8.08	1.42	−0.12	13 (4)*
Beaufort Sea^+^	9.16	13.70	0.66	0.24	26 (6)	14.0	16.6	0.84	−0.05	19 (6)*
Greenland Sea^+^	3.12	8.46	0.37	−0.16	31 (9)	8.95	8.40	1.06	0.06	17 (5)*
Central Arctic Ocean	15.10	16.84	0.90	0.23	22 (5)	17.4	20.5	0.85	0.15	22 (5)*
Europe^+^	−0.80	17.36	−0.05	−0.53	104 (33)	2.34	10.8	0.21	0.03	52 (13)
N.America^+^	8.26	19.05	0.43	−0.25	27 (8)	21.6	24.4	0.88	−0.15	17 (5)*
Greenland^+^	8.29	11.25	0.74	−0.13	20 (6)*	7.74	10.7	0.72	−0.30	18 (6)*
Siberia^+^	15.16	24.91	0.61	−0.23	22 (7)*	20.2	23.1	0.88	0.04	20 (5)*
Eurasia^+^	14.07	36.80	0.38	−0.44	26 (8)	21.8	29.8	0.73	0.08	23 (6)

*Note*. The TTE is the mean number of years needed for a trend emerge from 400 synthetic time series based on the trend, standard deviation, and autocorrelations. The standard deviation of TTE from the synthetic ensemble is given in parentheses. Trends that have emerged in the current observational record are noted with *. Regions are noted with a ^+^ if all‐sky and clear‐sky mean TTE are statistically different using a student’s t‐test with *p* <0.05.

Over both land and ocean, clouds damp SW_*acc*_ trends by more than a third and SW_*acc*_ standard deviations by almost 20%. The signal‐to‐noise ratio (SNR), defined as the magnitude of the trend in SW_*acc*_ divided by its standard deviation, is a measure of interannual variations; SNR quantifies the strength of secular trends relative to the underlying natural variability present in all climate records. Clouds decrease the SNR over both land and ocean since they reduce trends more than interannual variations. Since trends are more difficult to detect from noisier time series (Weatherhead et al., 1998), clouds effectively reduce the *detectability* of SW_*acc*_ trends. To quantify this effect, we estimate the expected number of years needed to detect a trend with 95% confidence, referred to as the time to emergence (TTE). Over the ocean, clouds increase the TTE by more than a third from 11 ± 3 to 16 ± 4 years. Clouds have a smaller impact on the TTE of SW_*acc*_ trends over land, increasing the TTE from 17 ± 5 to 19 ± 6 years. Although all‐sky SW_*acc*_ SNR is substantially smaller than clear‐sky SNR over land, autocorrelations impede trend detection in clear‐skies. Anomalies in clear‐sky SW_*acc*_ tend to persist in time over land resembling trends, and a longer record of observations is needed to discern such propagating natural variations from secular trends in the data set. The opposite is true in all‐sky SW_*acc*_ over land where negative autocorrelations are observed: anomalies are more likely to be followed by an anomaly of the opposite sign, leading to the earlier emergence of a trend, all else being equal. These competing behaviors close the gap between clear‐sky and all‐sky TTE over land, although the physical reasons for these autocorrelations are not entirely clear. Furthermore, the sample size for measuring 1‐lag autocorrelation here is relatively small and the corresponding uncertainties are not insignificant, ±0.44.

Taken together, the TTE of trends in total all‐sky Arctic SW_*acc*_ is 16 years. This is a significant result: the sea ice and snow cover losses shown in Figures S2c and S2d in Supporting Information S1 have now had a discernible impact on the amount of SW energy absorbed in the Arctic that emerged from natural variability in the last two decades of the CERES observational record. As a result, the current 21‐year observational record is now long enough to provide a robust test of predicted trends in this key driver of Arctic climate change in climate models despite conventional wisdom that a minimum of 30 years is needed to determine forced climate trends.

**Figure 3:**
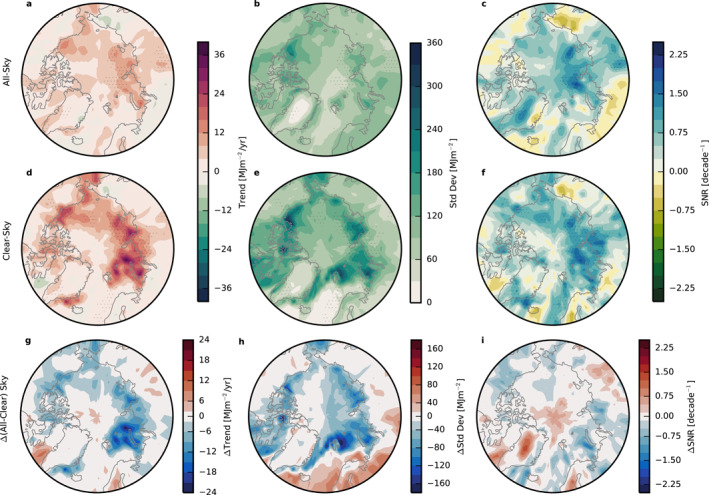
Accumulated shortwave trends (a and c), standard deviations (b and e), and signal to noise ratios (SNR) (c and f) calculated with all‐sky (a–c) and clear‐sky (d–f) fluxes over 2000–2020. Differences between all‐sky and clear‐sky conditions are shown in (g–i). SNR is calculated by dividing the trend by the standard deviation. Stippling represents grid boxes where trends have emerged in the observational record with 95% confidence.

While the total accumulated SW absorption over the Arctic provides a useful measure of how ice and snow cover losses have influenced the energy balance of the Arctic system as a whole, changes in absorbed solar radiation have local impacts enhancing regional melting and increasing SST in locations of sea ice loss, for example, Timmermans et al. (2018); Long and Perrie (2017). Recent declines in sea ice and snow cover exhibit distinct spatial patterns (Figures S2c and S2d in Supporting Information S1) that induce strong regional variations in the resulting SW_*acc*_ responses (Figure 3).

The largest trends under clear‐sky conditions correspond to areas with the greatest sea ice loss over 2000–2020 (Figure S2c in Supporting Information S1), for example the Barents, Kara, and Beaufort Seas. In these marginal seas, clear‐sky SW_*acc*_ trends are on the order of 20–30 MJm^−2^/yr, but reach a maximum of almost 40 MJm^−2^/yr in the Kara Sea. Consistent with Figure 2, trends are generally lower over land masses, with the greatest SW_*acc*_ trends observed over Northern Canada, approximately 20 MJm^−2^/yr without clouds. The few areas with negative SW_*acc*_ trends in Figure 3 correspond to regions that have increasing sea ice (Labrador Sea) or snow cover (northeast and northwest coasts of Russia) (Figure S2c in Supporting Information S1). Regions without sea ice or snow cover during March through September, including much of the Atlantic Ocean, have negligible trends.

Clouds decrease the magnitude of SW_*acc*_ trends by roughly half over both land and ocean (Figure 3g). Additionally, clouds reduce the area with statistically significant trends to half that in clear skies. Clouds lower the trends around the Barents and Kara Seas by upwards of 20 MJm^−2^/year. Clouds also weaken the magnitude of the SW_*acc*_ trend in the Labrador Sea, west of Greenland, where clear‐sky SW_*acc*_ is decreasing because sea ice is slightly increasing.

Large apparent trends over marginal seas do not, however, automatically guarantee rapid identification since natural variability also tends to be large. Over regions with seasonal sea ice cover, SW_*acc*_ standard deviations reach upwards of 300 MJm^−2^, approximately twice as large as the variability over most land surfaces. The exception to this ocean‐land contrast occurs over the Canadian Archipelago, that includes both snow and sea ice. Since regions with the largest trends also experience the largest variability, the SNR is critical for establishing the significance of trends relative to natural variations. High SNR provides a good indication of where trends are statistically significant with 95% confidence, indicated with stippling in Figures 3a–3f.

In spite of their high year to year variability, the marginal seas exhibit large SNR under clear‐sky conditions. Clouds not only reduce SW_*acc*_ over the ocean, but also exert a strong influence on its variability, especially on regional scales. In fact, while clouds reduce variability overall in Figure 2, two distinct regimes emerge in Figure 3h: clouds decrease variability over areas with seasonal or perennial sea ice but increase variability over areas that typically remain ice free, namely the North Atlantic. Over ocean regions that experience seasonal ice loss, clouds reduce the variability of SW_*acc*_ by roughly half, upwards of 150 MJm^−2^ in the Barents Sea and Canadian Archipelago. When present, clouds can increase the albedo over open ocean, but they also increase SW_*acc*_ variability because they are transient while open ocean has low and consistent albedo by comparison. Clouds therefore increase the standard deviation of SW_*acc*_ by about +75 MJm^−2^ relative to clear skies over open ocean. On the other hand, clouds have the opposite effect over areas with changing sea ice. While clouds are not always present, they persist in time for sufficiently long periods to dampen the albedo contrast between sea ice and open ocean, in turn reducing SW_*acc*_ variability.

As a result of these spatial variations, integrating SW_*acc*_ over all land and ocean areas conceals large regional differences in SNR and the TTE of trends in absorbed SW radiation that may have important local implications. Across individual marginal seas, for example, SW_*acc*_ trends vary by over a factor of two without clouds and a factor of four with clouds (Table 1). Over most marginal seas, all‐sky trends are roughly half of their clear‐sky counterparts. The only exception is the Barents Sea where the all‐sky trend is about a quarter of that in clear‐skies trend because of persistent cloud cover (Liu et al., 2012). The impact of clouds is less consistent over distinct land regions. SW_*acc*_ trends over North America, Siberia, and Eurasia are diminished by clouds, but the SW_*acc*_ trend is slightly increased by clouds over Greenland where cloud cover and snow cover have decreased in concert along the northeast edge of the ice sheet (Hofer et al., 2017).

Over much of the Arctic Ocean, the primary impact of clouds on SW_*acc*_ is to lower the SNR and, in turn, increase the time needed to detect trends. Clear‐sky SW_*acc*_ trends have emerged in the CERES‐EBAF record over all marginal seas (Table 1), but clouds have masked those trends from being statistically significant for half of the marginal seas (Barents, Beaufort, Greenland, and Central Arctic). Trends in the Laptev, Kara, and East Siberian Seas are statistically significant with 17–20 (±4–5) years of observations, a relatively short time period, and the SW_*acc*_ in the Chukchi Sea is also statistically significant with an average TTE of 22 (±6) years. Clouds have increased the TTE over these seas by 1–9 years. However, in the Barents Sea where sea ice trends are large, ubiquitous cloud cover reduces the local SW_*acc*_ trends much more than the interannual variability, decreasing the SNR (Figures 3g–3i) and substantially increasing the TTE (Table 1). Based on the measured trends and corresponding variability, clouds are also expected to delay the time needed to detect trends in the Beaufort Sea and Greenland Sea by 7 and 14 years, respectively, enough to mask trends that would otherwise have been detectable in the current satellite record. Clouds have little effect on the estimated time required to detect SW_*acc*_ trends in the central Arctic Ocean where perennial sea ice persists (Figure 3), although the clear‐sky SW_*acc*_ trend is statistically significant under observed clear‐sky conditions but not all‐sky.

There are also two distinct regions of cloud impacts on absorbed solar radiation over land. Clouds impact North America in a similar manner as over the marginal seas, reducing SW_*acc*_ trend, SNR, and increasing the TTE. Based on Figure 3g, clouds decrease SW_*acc*_ trends over continental North America with less impact on their variability. From June through September most land surfaces contribute little to the TOA albedo (Sledd & L’Ecuyer, 2019), so the transient nature of clouds can again provide intermittent contrast to the relatively dark surface albedo. While the SW_*acc*_ trend has emerged over North America, clouds have delayed its detectability beyond the current CERES‐EBAF record. This is true along the coast of Eurasia as well, but SW_*acc*_ trends have not emerged over Europe, Siberia, or Eurasia.

## 4 Discussion and Conclusions

The 21 year record from CERES is now long enough to have definitively measured recent increases in total all‐sky absorbed SW radiation in the Arctic. While clouds generally reduce the magnitude of SW_*acc*_ trends and increase the number of years required to measure a trend relative to a clear‐sky scenario, sea ice and snow cover have declined sufficiently that their impacts on absorbed solar radiation have emerged in the two decade observational period, in both clear‐sky and all‐sky conditions. Although 21 years is a short observational period for trend detection, previous work has found that sea ice has already declined so much in the satellite era that it is significantly different than preindustrial conditions during this time period (Landrum & Holland, 2020). However, even though sea ice largely determines the pan‐arctic surface albedo, it was not obvious that such changes directly manifest themselves in the TOA energy budget due to the substantial influences of intervening clouds. This work demonstrates that the solar energy input into the Arctic has definitively increased as a result of these sea ice losses over the modern satellite era.

Clouds have, however, masked SW_*acc*_ trends from emerging over half of the marginal seas. These findings generally agree with previous work in terms of where the greatest SW absorption trends have been observed. For example, significant trends have been consistently observed in the Pacific sector of the Arctic Ocean, particularly in the Beaufort Sea (Hartmann & Ceppi, 2014; Perovich et al., 2007), although the magnitude of those trends is not always consistent (Wu et al., 2020). Our work shows greater trends in the Kara and Laptev Seas compared to others, for example, Perovich et al. (2007), although they agree with spatial patterns during the spring and early summer when there is significant incoming SW (Letterly et al., 2018).

While earlier work found clouds had a limited impact on SNR when evaluated over the entire Arctic, we expose a more nuanced picture. As in Sledd and L’Ecuyer (2021), clouds reduce the magnitude of trends even on regional scales, but cloud influences on SW_*acc*_ interannual variability fall into two distinct regimes. Clouds decrease variability where the surface changes during the melt season, such as the marginal ice zone, but they increase variability wherever the surface has a consistent albedo, such as open ocean in North Atlantic. This suggests pan‐arctic studies may inadvertently miss important regional differences that are critical for defining local surface and temperature responses.

Because clouds account for the majority of the TOA albedo in the Arctic (Sledd & L’Ecuyer, 2019), changes in cloud cover impact SW absorption, (e.g., Alkama et al., 2020). Previous studies using passive sensors have found small (Comiso & Hall, 2014; Letterly et al., 2018) or no (Choi et al., 2020) cloud cover trends in summer over the Arctic. However, passive sensors underestimate cloud fraction over bright, cold surfaces compared to active sensors (Chan & Comiso, 2013), leading to biases in trend estimates as sea ice declines (Liu et al., 2010). Using active sensors, no statistically significant trend in Arctic cloud cover has been determined (Kay et al., 2016), possibly owing to the relatively short length of this satellite record (2006‐present). Ultimately, longer data records are needed to determine how clouds are changing in the Arctic and the consequences for SW absorption.

Of the regions where trends have not yet emerged, many are predicted to emerge in the next decade. The continuity of satellite‐based radiation budget measurements over the Arctic will be critical for determining if and when such trends emerge and to continue monitoring the impacts of climate change in the Arctic.

## Supporting information

Supporting Information S1

## Data Availability

ArORIS data are available at the CloudSat Data Processing Center: http://www.cloudsat.cira.colostate.edu/community-products/arctic-observation-and-reanalysis-integrated-system. Original CERES‐EBAF data are available at https://ceres.larc.nasa.gov/data/. The NSIDC provides data for sea ice (https://doi.org/10.7265/N59P2ZTG) and snow cover (https://doi.org/10.5067/MODIS/MOD10CM.006). Code used for the time to emergence analysis in this study is available at Zenodo via https://doi.org/10.5281/zenodo.5644382.
