# Supplementary material for: Emerging Trends in Arctic Solar Absorption
Source: Geophys Res Lett. 2021 Dec 24;48(24):e2021GL095813. doi: 10.1029/2021GL095813 (PMC9285040; doi:10.1029/2021GL095813)
Supplement: Supplementary file 1 — Supporting Information S1 [file GRL-48-0-s001.pdf]

Supporting Information for Emerging Trends in Arctic Solar Absorption

A. Sledd[1]and T. S. L'Ecuyer[1,2]

[1]University of Wisconsin-Madison

[2]Cooperative Institute for Meteorological Satellite Studies

Contents of this file

1. Figure S1
2. Figure S2

Introduction Supplementary figures referenced in main text.

References

Christensen, M. W., Behrangi, A., L'ecuyer, T. S., Wood, N. B., Lebsock, M. D., & Stephens, G. L. (2016). Arctic observation and reanalysis integrated system: A new data product for validation and climate study. *Bulletin of the American Meteorological Society*, *97*(6), 907–916.

Hall, D. K., & Riggs, G. A. (2015). *MODIS/Terra snow cover monthly L3 global 0.05deg CMG, version 6.* Retrieved from https://doi.org/10.5067/MODIS/MOD10CM.006

Peng, G., Meier, W. N., Scott, D., & Savoie, M. (2013). A long-term and reproducible passive microwave sea ice concentration data record for climate studies and monitoring. *Earth System Science Data*, *5*(2), 311–318.

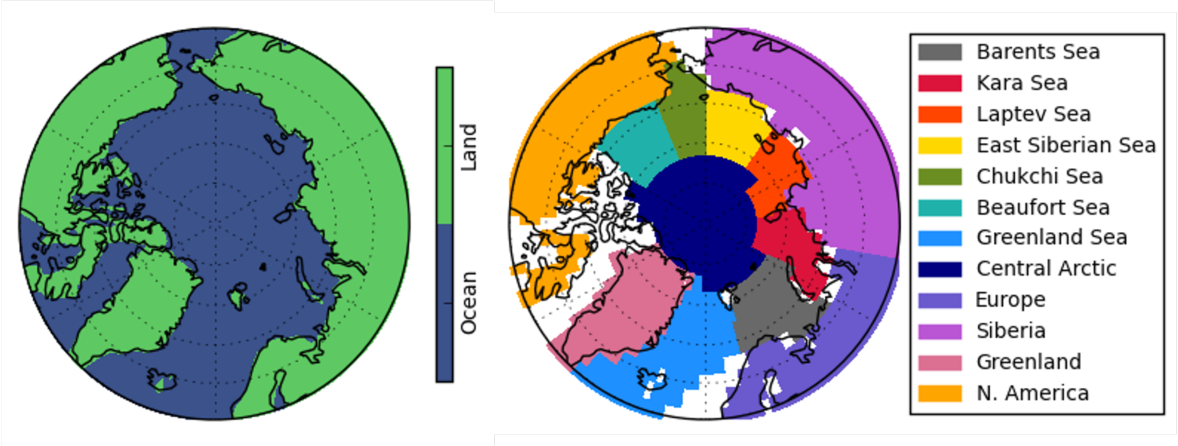

Figure S1. Maps of Arctic regions. (left) Area considered land or ocean in the Arctic based on regridded land fraction variable from NCEP reanalysis. (right) Individual land areas and seas used in the text. Eurasia is the total area encompassing Europe and Siberia. NCEP land masks from the Arctic Observation and Reanalysis Integrate System (ArORIS) (Christensen et al., 2016) are also used to determine the individual land regions. Marginal seas are based on the Multisensor Analyzed Sea Ice Extent regions from the National Snow and Ice Data Center interpolated to the ArORIS grid.

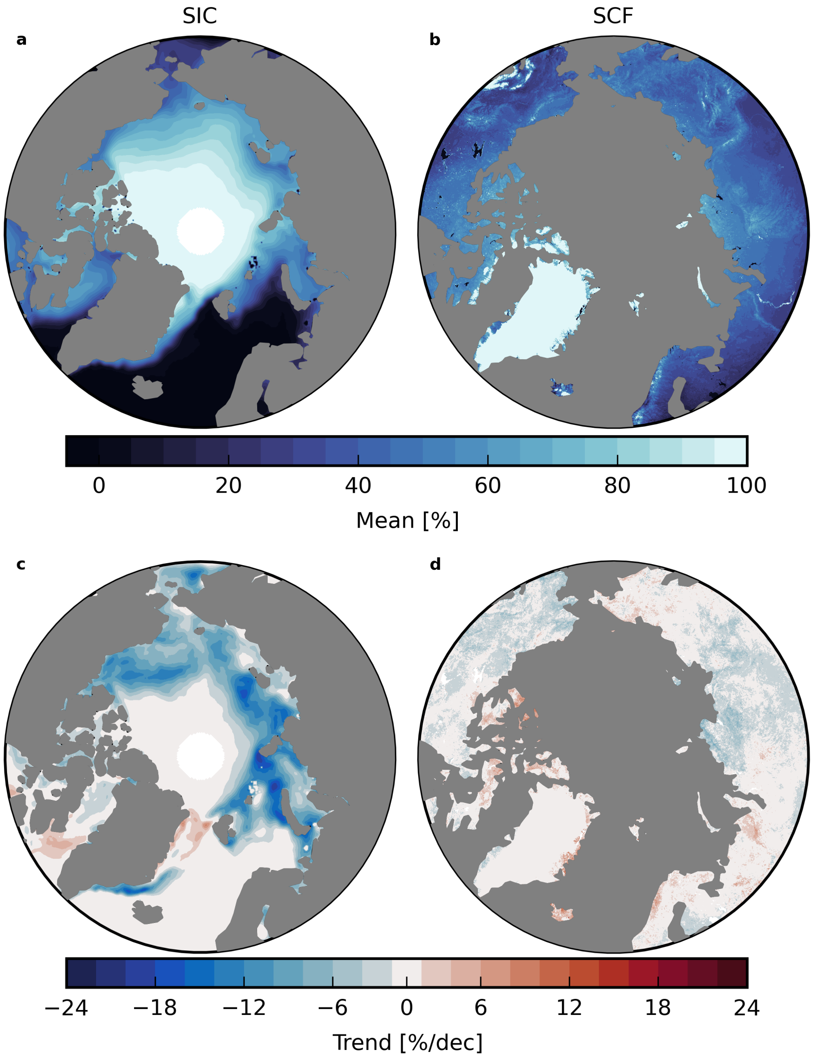

Figure S2. Average sea ice concentration (SIC) and snow cover fraction (SCF) for March through September from 2000-2020 (a,d) and corresponding trends (c,d). Trends are calculated using linear least-squares regression. SIC is from the NOAA/NSIDC Climate Data Record of Passive Microwave Sea Ice Concentration Version 3 that uses passive microwave radiometers, the Special Sensor Microwave Imager and the Special Sensor Microwave Imager/Sounder, from the Defense Meteorological Satellite Program (Peng et al., 2013). SIC in this dataset is derived from a combination of the NASA Team and Bootstrap algorithms. We use SCF from version 6 of the MODIS/Terra Snow Cover Monthly dataset with 0.05 degree resolution of Climate Modeling Grid cells, based on Normalized Difference Snow Index snow cover algorithm (Hall & Riggs, 2015).
